# Projection-Based Simulation Method for Robotic 3D Printing of Large-Scale Polymer Composite Structures

**DOI:** 10.3390/polym17111564

**Published:** 2025-06-04

**Authors:** Yuen Xia, Kil-Sung Lee, Sung Kyu Ha

**Affiliations:** 1 Department of Mechanical Engineering, Hanyang University, 222 Wangsimri-ro, Seonjong, Seoul 04763, Republic of Korea; xiayuen@hanyang.ac.kr; 2 Human Composites Co., Ltd., 152, Jayumuyeok-ro, Gunsan-si 54002, Jeollabuk-do, Republic of Korea; ks2994@humancomposites.com

**Keywords:** large structure 3D printing, fused deposition modeling (FDM), short fiber-reinforced polymer composites, characterization of material properties, projection method

## Abstract

As large-scale additive manufacturing advances, the reliable prediction of the structural behavior of FDM-printed composites is becoming increasingly important. However, existing finite element methods often oversimplify the material anisotropy introduced by the printing path. This study proposes a projection-based method that maps toolpath-defined fiber orientations directly into a finite element model to represent anisotropic mechanical behavior. The mechanical properties of printed carbon fiber-reinforced ABS were experimentally characterized in three directions (UDL, UDT, and UD10). The results confirmed strong anisotropy, with elastic moduli ranging from 3.2 to 9.8 GPa and tensile strengths from 20 to 81 MPa. The shear modulus and strength obtained from the 10° off-axis tensile tests were 1.17 GPa and 10.9 MPa, respectively. This directional data enabled the implementation of the FE model of a 20 m-long printed ship structure. The predicted mid-span deflection (2.19 mm) differed by only 5% from the experimental measurement (2.08 mm). While effective, this method may face challenges with highly irregular geometries. Nevertheless, it offers a scalable approach for the accurate simulation of FDM-printed composites.

## 1. Introduction

**Figure 1:**
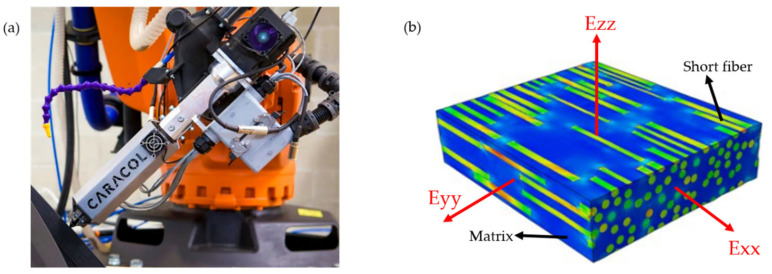
FDM fabrication process and anisotropy of 3D-printed materials: (**a**) Robot Extruder [41]; (**b**) Anisotropy of 3D-Printed materials.

With the advancement of 3D printing technology, the field has gradually transitioned from small-scale fabrication to the production of large and complex structures [1]. Within this evolving landscape, fused deposition modeling (FDM) has emerged as one of the most extensively employed additive manufacturing techniques for large-scale structural production, primarily owing to its simplicity and cost-effectiveness [2]. The printing process is fully automatic using a robotic system and an extruder with feeding of short-fiber-reinforced pellets, as illustrated in Figure 1a.

Recently, short-fiber-reinforced thermoplastic composites have emerged as a central research focus in material extrusion additive manufacturing. Short-fiber-reinforced thermoplastic composites for material-extrusion additive manufacturing typically employ E-glass, carbon, aramid, basalt, or natural lignocellulosic fibers, such as flax and hemp [3,4,5,6,7,8,9]. Glass fibers remain the most widely used owing to their low cost and balanced tensile strength (≈1.5 GPa); however, their relatively high density (~2.55 g cm^−3^) limits weight savings in lightweight structures [3,4]. Basalt fibers offer slightly higher modulus and superior thermal stability but have a density comparable to that of glass and a less established supply chain [5,6]. Aramid fibers (e.g., Kevlar^®^) provide excellent impact resistance and high specific strength but suffer from moisture uptake and a lower compressive modulus, which complicate printing and structural design [7,8]. Natural fibers are attractive for sustainability; however, their low thermal resistance (<200 °C) and variability hinder their integration with engineering thermoplastics processed at >250 °C [9]. In contrast, carbon fibers combine the highest specific modulus (≈130 GPa g^−1^ cm^3^) and tensile strength (>3 GPa) with a low density (~1.8 g cm^−3^), outstanding thermal stability, and intrinsic electrical conductivity that promotes inter-filament fusion during laser- or Joule-assisted printing [10,11]. For these reasons, and because chopped carbon fiber feedstock is now commercially available for fused deposition modeling, carbon fiber was selected as the reinforcement in the present study.

Many researchers have shown that adding short carbon fibers markedly improves the stiffness and strength compared with neat polymers. For example, Ivey et al. [12] reported a 150% increase in the tensile modulus of short carbon fiber/nylon composites and an approximately 140% increase in the elastic modulus of carbon fiber/PLA composites after annealing. Similarly, Gupta and Fidan [13] demonstrated that increasing the fiber volume fraction in short carbon fiber/PP filaments substantially improved Young’s modulus along the printing direction, albeit with only slight gains in the transverse direction, highlighting the pronounced anisotropy of these materials. Despite these improvements, critical challenges such as fiber breakage, void formation, and associated strength persist [3,14,15]. Thus, although short-fiber-reinforced thermoplastic composites offer considerable promise for structural applications, their anisotropic mechanical behavior and microstructural heterogeneities complicate accurate performance prediction and design. Consequently, this study aims to develop and experimentally validate a projection-based finite-element workflow that links recorded tool paths to element-wise fiber orientations, enabling an accurate simulation of large FDM polymer-composite structures.

The anisotropic mechanical behavior of short-fiber-reinforced thermoplastic composites fundamentally stems from the type, morphology, distribution, and orientation of embedded short fibers. As illustrated in Figure 1b, unlike traditional single-component homogeneous materials, short-fiber-reinforced thermoplastic composites exhibit a complex hierarchical microstructure that induces pronounced anisotropy across the macro-, meso-, and microscales. In particular, the molten matrix flow during material extrusion induces fiber alignment predominantly along the printing path, resulting in enhanced stiffness and strength in the longitudinal direction, while comparatively inferior properties develop transversely. This directional dependency is often modeled using a transverse isotropic framework.

Given the critical influence of fiber alignment on mechanical performance, the accurate characterization of direction-dependent properties is essential for the reliable design and simulation of large-scale 3D-printed structures. However, despite extensive research efforts investigating the effects of infill angle, raster orientation, and processing parameters, the current experimental databases remain insufficient to provide a complete set of anisotropic material constants required for finite element modeling [16,17,18,19,20]. To address this limitation, the present study proposes a systematic material testing protocol capable of capturing the full suite of anisotropic properties of short-fiber-reinforced thermoplastic composites, thereby bridging the crucial gap between experimental characterization and computational simulation.

Robotic inclined-surface printing has emerged as an effective manufacturing strategy for fabricating large-scale, free-form components. Shajay Bhooshan et al. [21] developed the Print-Path Design toolchain, which combines functional-surface representation with six-axis synchronized interpolation to enable support-free deposition on planes inclined at 35–45 degrees; using this method, they fabricated 53 concrete arch blocks for a footbridge with a 16 m span. Nadal Serrano [22] integrated a 25 kg/h pellet extruder with a KUKA KR-120 robot and established a design-to-manufacture workflow that enables rapid iteration of 3 m-scale polymer prototypes. Georgiou et al. [23] combined variable formwork with a six-axis robot to print cement-based material on inclined “I-, S-, U-, and W-shaped” surfaces, demonstrating both the feasibility and the practical limits of an adaptive formwork–robot synergy for prefabricating complex curved structures. Luu et al. [24] built an FDM 3D printing system around a six-DOF industrial robot and achieved support-free continuous deposition on surfaces inclined up to 80°, further demonstrating that multi-axis path planning and coordinated robot–extruder control is critical for high-angle printing. Hai-ming Zhao [25] developed the Inclined Layer Printing (ILP) technique, in which the build platform—rather than the robot arm—is tilted. This strategy enables support-free fabrication of features with up to 60° overhangs and reduces support material consumption by approximately 87%. Collectively, these studies highlight the critical role of multi-axis path planning and coordinated robot–extruder control in achieving complex, large-scale additive manufacturing.

**Figure 2:**
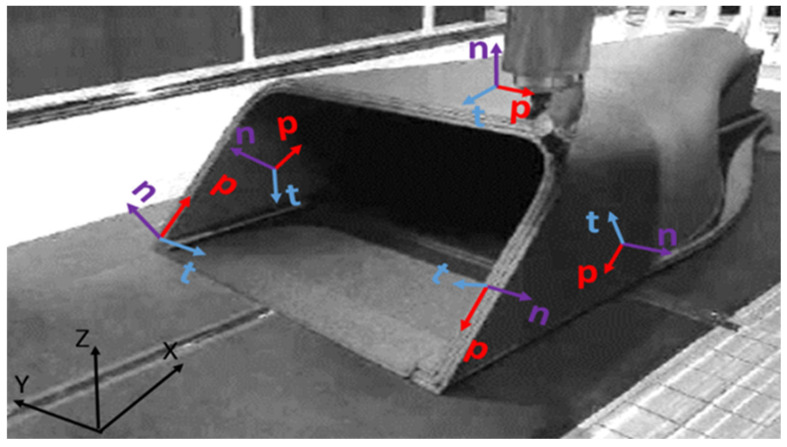
Local material coordinate systems in 3D-printed structures.

As the scale and complexity of 3D-printed structures continue to grow, the limitations of conventional planar 3D printing with fixed angles of 0° or 90° have been increasingly exposed. When large-scale components with pronounced overhangs or intricate geometries are fabricated, conventional planar printing methods inevitably require extensive support structures, significantly increasing material consumption, prolonging printing duration, and complicating post-processing operations. In contrast, robotic inclined-surface printing significantly mitigates these issues by enabling support-free deposition, thereby reducing material waste, shortening production cycles, and improving design flexibility. These advantages have led to their growing adoption in industries such as shipbuilding and mold manufacturing, where lightweight, corrosion-resistant polymer composites are preferred for their structural and economic benefits [1,26,27,28,29,30,31,32,33,34,35]. Nevertheless, the layer-by-layer deposition characteristic inherent to robotic inclined-surface printing introduces pronounced anisotropy within the printed structures, posing new challenges for the prediction of mechanical performance. As illustrated in Figure 2, variations in the printing direction P give rise to distinct material orientations on different faces of the structure; the printing direction P and thickness direction T on each printed surface are rotated about the normal direction N. Consequently, each face of the same component exhibits unique mechanical properties, thereby imposing higher demands on subsequent mechanical analysis and structural optimization. Although simulation methods for 3D-printed structures have been extensively investigated in previous studies [36,37,38,39,40], in which various numerical techniques and computational models to predict their mechanical behavior were developed, these methods predominantly focus on global mechanical properties or interlayer bonding strength and fail to comprehensively capture the influence of the printing path on the ultimate structural performance, representing a notable gap in the field of 3D-printing mechanical simulations. Accordingly, this study proposes a projection-based approach to accurately capture the anisotropic behavior of large-scale polymer composite structures fabricated using FDM. By mapping local fiber orientations based on the actual printing path, this method allows for the precise incorporation of directional material properties into finite element simulations.

To address the challenges associated with anisotropy in FDM-printed polymer composites, this study introduces a projection-based method for accurately analyzing the mechanical behavior of large-scale printed structures. This method defines local fiber orientations directly from the actual printing path, enabling a more precise representation of the anisotropic properties in finite element simulations. To support the projection method, we established a systematic material characterization workflow in which direction-dependent mechanical properties were characterized through tensile tests in three orientations (UDL, UDT, and UD10), with the latter capturing the shear response of the material. Implemented in ABAQUS, the proposed methodology was applied to simulate a 3D-printed ship mockup, and the numerical predictions of ship deflection agreed with full-scale measurements within 5%, demonstrating the method’s accuracy and engineering applicability.

The remainder of this paper is organized as follows: Section 2 describes the material preparation and directional characterization. Section 3 presents the projection algorithm and numerical validation. In Section 4, the method is applied to a twenty-meter ship mockup. Section 5 compares the simulated and experimental deflections, and Section 6 concludes with the limitations and future work.

## 2. Characterization of Printed Materials

### 2.1. Material Preparation

In this work, an ABS/CF20 polymer composite material manufactured by Airtech International Inc. (Huntington Beach, CA, USA) was utilized as the matrix. During the manufacturing process, a high-capacity extruder capable of depositing the material at approximately 150 lb h^−1^ was utilized, coupled with a nozzle diameter of 10 mm; each layer featured a width of approximately 18.75 mm and a thickness of 3 mm, with the printing orientation set at 45°. To produce the composite panels required for experimentation, FDM was employed using uniform processing parameters (including printing temperature, nozzle diameter, extrusion speed, and layer thickness), thereby forming sufficiently large plates. In the RoboDK v5.7.4 (RoboDK Inc., Montreal, QC, Canada), a 45° printing strategy was adopted by setting the substrate orientation to a 45° angle relative to the build platform reference. This approach provided robust experimental data to support the subsequent simulation analyses of a 45°-printed ship.

### 2.2. Specimen Preparation

**Figure 3:**
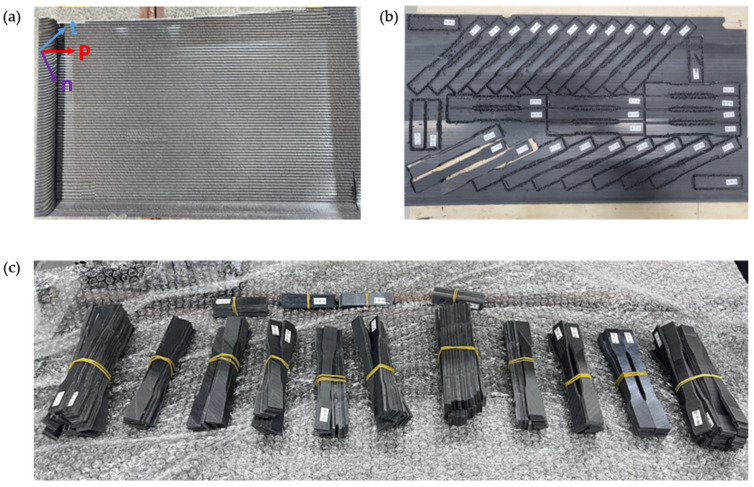
3D-printed specimens (UDL, UDT, UD10): (**a**) 3D printed panel; (**b**) Milling & CNC process; (**c**) 3D printed specimens.

To ensure accurate specimen fabrication, dimensional precision, and surface quality, a CNC precision machining device was initially used to cut the composite panels, with the cutting paths and dimensions strictly following the ASTM D638 guidelines [42] to produce standard-compliant dog-bone-shaped tensile specimens. As illustrated in Figure 3, based on the fiber orientation relative to the tensile loading direction, three types of specimens were designed and fabricated: UDL (0°, fibers parallel to the tensile axis), UDT (90°, fibers perpendicular to the tensile axis), and UD10 (10 deg-off, fibers oriented at a 10° angle to the tensile axis). The selection of fiber orientation angles in our study was based on both standard practices in composite testing and the need to capture distinct mechanical responses relevant to FDM-printed structures. UDL and UDT represent extreme loading conditions along the principal fiber direction and orthogonal to it, whereas UD10, which is situated between these two extremes, is a practical and effective means of characterizing the interlaminar shear properties of UD composite laminates by inducing shear through extension-shear coupling, and provides data representative of real-world mixed loading conditions [43]. The 10° off-axis test introduces coupled axial and shear deformation. Under the small-angle assumption, the shear strain is the dominant contributor to the axial response. This allows the in-plane shear modulus to be approximated using inverse laminate theory or micromechanics-based models. This approach is widely accepted and has been used in ASTM D3518 and D7078 standards, as well as referenced in the composite design literature (e.g., Daniel and Ishai, 2006) [44,45,46].

During the CNC cutting process, a constant spindle speed, controlled feed rate, and adequate cooling/lubrication were employed to minimize the thermal and mechanical impacts on the interlinear regions, thus preventing any noticeable heat damage or delamination on the specimen surfaces. Finally, the geometry, tolerance, and surface roughness of each specimen were rigorously maintained in strict accordance with the ASTM D638 requirements to ensure the reliability and reproducibility of all test data.

### 2.3. Mechanical Test

To systematically investigate the mechanical response of composite materials with different fiber orientations, a universal testing machine (manufactured by Jinan XLC Testing Machine Co., Ltd., Jinan, China) was employed to conduct uniaxial tensile tests on various specimens under ambient conditions. After each specimen was clamped, a preload of 5–10 N was applied to remove the seating slack, and the crosshead speed was fixed at 1 mm min^−1^. The load–displacement data were recorded continuously, and the strain was captured using gauges and a high-precision extensometer at 1 Hz. The key mechanical parameters, including the tensile strength, tensile modulus, and elongation at break, were derived from stress–strain curves. To evaluate the stability and repeatability of the test data for each specimen type (UDL, UDT, and UD10), at least three replicate tests were conducted for every group, and the mean values and standard deviations were calculated for further statistical analysis.

### 2.4. Data Analysis and Discussion

Based on the summary table and stress–strain curve, the ABS/CF20 composite fabricated in this study clearly has the highest strength and stiffness along the fiber direction (UDL), whereas its properties are the weakest when loaded perpendicular to the fibers (UDT). The UD10 specimens fell between these two extremes. This disparity primarily arises from the pronounced anisotropy caused by the orientation of the short carbon fibers within the polymer matrix: when the load is aligned with the fiber distribution (UDL), the high-strength and high-modulus carbon fibers can be fully utilized to increase the load-carrying capacity; conversely, when the load is perpendicular to the fibers (UDT), the fibers cannot effectively support the tensile load, resulting in a significant reduction in the overall mechanical performance. Because the UD10 orientation is only 10° from the principal fiber direction, fiber reinforcement is more effectively used than in the UDT case, thus exhibiting strength and stiffness higher than those for UDT but lower than those for UDL.

**Figure 4:**
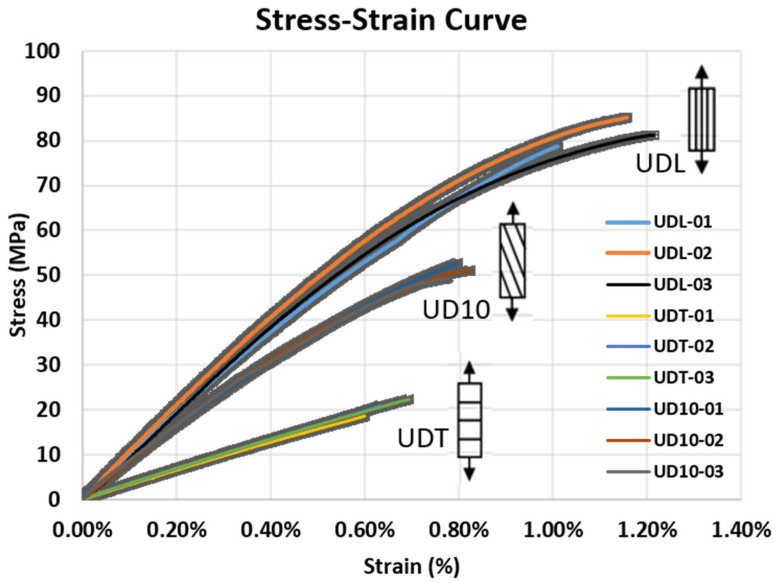
Stress–strain curves of short carbon fiber-reinforced ABS specimens tested in three orientations: UDL (0°), UDT (90°), and UD10 (10° off-axis).

From Table 1, the elastic moduli (E_x_ = 9.80 GPa, E_y_ = 3.23 GPa) and strengths (X = 81.1 MPa, Y = 20.2 MPa, S = 10.9 MPa) further quantify the degree of anisotropy: the elastic modulus in the principal fiber direction is approximately three times greater than that in the perpendicular direction, whereas the strength is nearly four times greater. Moreover, the shear modulus (G_xy_ = 1.17 GPa) and Poisson’s ratio (ν_xy_ = 0.32) were consistent with values typical of short carbon fiber-reinforced polymers, indicating a relatively uniform fiber dispersion and bonding within the matrix. Notably, the standard deviations for these parameters are low, reflecting the robust reproducibility and stability of the fabrication and test procedures. Similarly, the stress–strain curves in Figure 4 corroborate the influence of fiber orientation on the deformation behavior of the material: UDL specimens exhibit a higher initial stiffness slope and fail at a higher stress level, whereas UDT specimens reach fail at lower stress since the fibers cannot effectively carry a longitudinal tensile load. The UD10 specimens lie between these two extremes, demonstrating a progressive decline in strength and stiffness with increasing fiber misalignment, yet still retaining a degree of reinforcement. This gradual variation in the mechanical properties is especially significant for actual structural design, in which multiaxial or bending loads cause fibers at various angles to collectively bear the load.

Due to the nature of fused deposition modeling (FDM), variability in the mechanical testing results is expected and should be interpreted considering process-induced uncertainties. Several sources may have contributed to this variability. These include local fluctuations in the filament feed rate, nozzle temperature inconsistencies, variations in layer bonding quality, and minor deviations in the raster path owing to machine resolution or slicing artifacts. In particular, off-axis specimens (e.g., UD10) are more susceptible to such inconsistencies because shear-dominated deformation is more sensitive to weak interlayer adhesion and local-print heterogeneities. To mitigate these effects, all specimens were fabricated using tightly controlled printing settings, and the tensile data were averaged across at least three repetitions per orientation.

Overall, the experimental results clearly illustrate that fiber orientation plays a critical role in determining the final mechanical performance of short fiber-reinforced composites. By integrating the data from the UDL, UDT, and UD10 specimens, researchers can obtain a more comprehensive set of material parameters, enabling simulation analyses to more accurately capture the anisotropic characteristics and high-strength advantages offered by fiber reinforcement, which can be better leveraged in both optimization and real-world applications.

## 3. Implementation of Projection Method in Finite Element Analysis (FEA)

**Figure 5:**
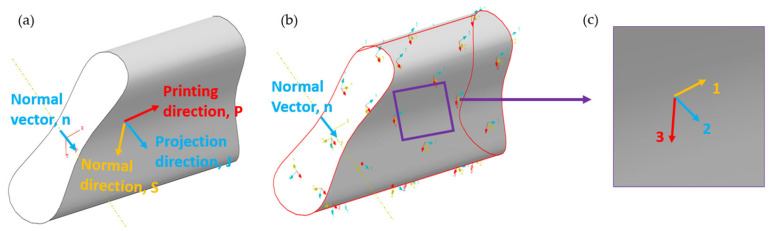
Schematic illustration of the projection-based method for determining local printing directions on inclined surfaces: (**a**) local coordinate system definition and surface normal direction on an inclined surface; (**b**) projection process used to decompose the normal vector into in-plane components and define the fiber alignment direction; (**c**) final assignment of a local coordinate system (Direction 1 = printing direction, Direction 2 = projection direction, Direction 3 = normal direction), used to represent anisotropic material orientation in the finite element model.

To precisely take into account the anisotropic properties of the 3D printed composites in the inclined planes, a computation procedure that can be implemented in Finite Element Analysis (FEA) is required. This method is called the Projection method since the local coordinates are projected onto the global coordinates, as shown in Figure 5.

### 3.1. Introduction of Projection Method

To accurately capture the anisotropic behavior of 3D-printed structures with inclined surfaces, defining the local material orientation based on the printing path is essential. As shown in Figure 5a, the process begins by identifying the normal vector *n* of the inclined printing surface and projecting it onto a reference plane to determine the projection direction J. Subsequently, the printing direction p is derived by combining projection direction J and normal direction S on the reference plane. Based on this method, a finite element model was established in ABAQUS, and the material orientations were defined accordingly. As shown in Figure 5b,c, a local coordinate system was defined on the inclined surface to represent the material anisotropy. In this system, Direction 1 is aligned with the printing direction p, Direction 2 corresponds to the projection direction j on the reference plane, and Direction 3 is defined as the surface normal vector n. This orthogonal basis was used to assign the local material orientations in the finite element model.

### 3.2. Derivation of Formulae

This vectorial formulation of the printing direction is an original contribution of this study. This projection-based interpretation, which directly links the surface normal and local tool-path orientation on inclined surfaces, has not been reported in the literature. This method was developed to provide a clear and geometrically consistent approach for defining the anisotropic material orientation in curved or multi-angled FDM structures. Compared with conventional methods that assign uniform or average fiber orientations to entire regions, the proposed projection-based method accounts for local geometric variations by aligning the element-wise material orientation with the actual printing path. This significantly improves the fidelity of the anisotropic behavior representation, especially for curved or inclined structures, and helps bridge the gap between the simulation and real-world printed performance.

To prove that the normal vector n of a 3D-printed surface is perpendicular to the printing direction P, we first define the normal S of the target surface and the projection J of n onto that surface. As illustrated in Figure 5a, the derivation proceeds as follows: the normal vector n on the inclined plane is decomposed into its component J, which is projected onto the other printed surface.

(1) $$n=J+\left(n\cdot S\right)\cdot S$$

where J is the projection of n onto the target surface, and S is the surface normal. Next, by applying the vector cross-product relationship

(2) J×S=P

The printing direction P is found to be orthogonal to both J and S. Furthermore, from

(3) P·S=0

P can be inferred to be perpendicular to S and J. Substituting these conditions back into Equation (1) ultimately yields

(4) P·n=n⊥P

The equation indicates that the surface normal n is perpendicular to the printing direction P. This process provides a clear geometric basis for defining both the printing path and direction on curved or inclined surfaces, thus ensuring that in subsequent numerical simulations or practical printing applications, the relationship between the printing surface and its normal is accurately identified.

To support the implementation of this directional projection method, we briefly clarify the material modeling assumptions used in this study. The term orthotropic applies to materials with fully independent stiffnesses in three orthogonal directions without any planar symmetry. In contrast, transversely isotropic materials have a unique axis (typically the printing direction), while the transverse plane exhibits isotropic properties. Since the primary concern of this work is the in-plane behavior of the printed layers, we assumed that the properties in the z-direction (thickness) are the same as those in the y-direction, leading to a transversely isotropic approximation. This assumption simplifies the material definition while capturing the primary anisotropy induced by the FDM process.

**Figure 6:**
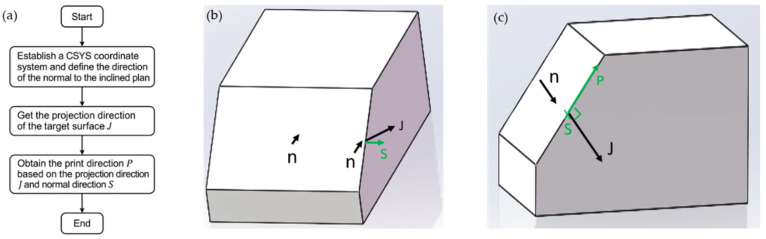
Illustration of the procedure for determining the printing direction based on inclined surface geometry. (**a**) Flowchart of projection implementation in FEA software (Abaqus 2017, Dassault Systèmes, Providence, RI, USA). (**b**) Projection of the normal vector n of the inclined surface to obtain J, with S denoting the inclined surface normal. (**c**) Determination of printing direction P using the cross product of J and S.

To illustrate the geometric implementation of this method, the complete procedure for determining the printing direction P on an inclined surface is shown in Figure 6. As shown in Figure 6a, the process begins with defining the surface normal n of the inclined face within a local coordinate system. This workflow can be directly implemented in Abaqus to automatically assign element orientation vectors. The normal n is then projected onto the inclined surface to obtain vector J, which lies entirely within the surface and satisfies the orthogonality condition J·S = 0, as shown in Figure 6b. Finally, as shown in Figure 6c, the printing direction P is calculated as the cross product of J and surface normal S, ensuring that P remained tangent to the surface and perpendicular to both J and S. This geometric procedure provides a robust framework for the numerical simulation of large-scale 3D structures.

While the proposed projection-based approach enables the effective mapping of element-wise fiber orientations on inclined surfaces, some limitations must be acknowledged. First, the method assumes relatively smooth geometries with well-defined local normal and coordinate systems. Its application to highly irregular or doubly curved surfaces may require additional treatments, such as local mesh refinement or curvature-aware smoothing. Second, the framework assumes consistent print quality and uniform deposition across the printed part, which may not be valid under varying machine conditions or material inconsistencies. Finally, the current formulation relies on a transversely isotropic material assumption; generalization to orthotropic or heterogeneous multi-material systems requires further development. Despite these limitations, the approach provides a robust foundation for practical implementation and can be extended to more complex scenarios in future work.

### 3.3. Method Validation

**Figure 7:**
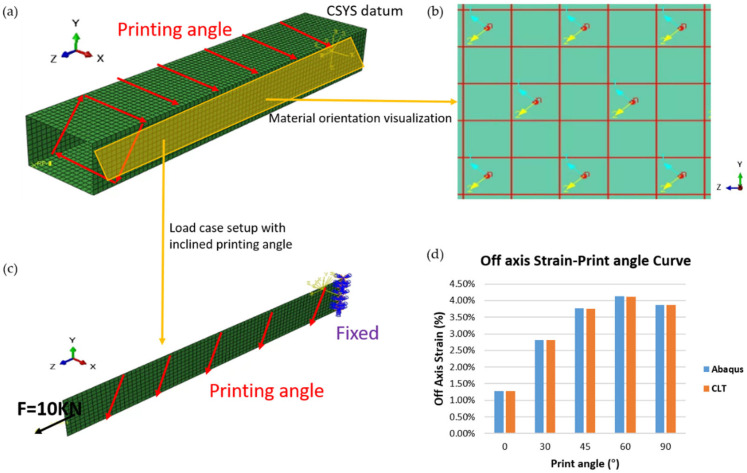
Method validation using a simplified quadrilateral box model to assess the accuracy of the projection-based material orientation assignment: (**a**) meshed box with inclined printing angle and definition of local coordinate system (CSYS); (**b**) projected material orientation vectors assigned to side faces, visualized to confirm local alignment consistency; (**c**) application of boundary conditions: a 10 kN inclined point load is applied at one end, while the other is fixed; (**d**) comparison between simulated off-axis strain results (Abaqus) and theoretical predictions (CLT) across different print angles. The simulation was performed using the Abaqus 2017 (Dassault Systèmes, Providence, RI, USA).

The quadrilateral box model in Figure 7 illustrates how the projection method assigns element orientations and verifies its accuracy with respect to classical laminate theory (CLT) [47]. First, to emulate a structure printed on an inclined plane, we constructed a shell-element model of the quadrilateral box. For each face of the model, we assigned a specific printing direction consistent with the actual printing process. As shown in Figure 7a,b, the projection method was used to project the desired printing directions onto the model surfaces, ensuring that Direction 2 was precisely aligned with the intended printing direction. The mesh resolution used in this study was set to 50 mm × 50 mm, which reflects a practical and commonly adopted element size in the finite element modeling of large-scale FDM structures [48,49]. This mesh density offers a balance between computational efficiency and sufficient resolution of the global deformation response. Although a full mesh convergence study was not performed, this mesh size has been used in previous studies involving polymer-based additive manufacturing of meter-scale components, where computational tractability is essential.

Taking the 45° printing angle as an example, we observed that the defined material coordinate system matches the target printing orientation, thereby guaranteeing that the numerical analysis captures the actual fiber trajectories and material anisotropy inherent to the printing process. However, if loads are directly applied to the entire structure, including both the top and bottom faces, complex boundary effects and stress paths would ensue, complicating a direct comparison with theoretical predictions. To circumvent these confounding factors, we extracted only the portion of the shell element that featured an inclined printing angle, as shown in Figure 7c. We then fixed one end of this segment and applied a tensile load of 10 KN to the opposite end so that the mechanical response of the inclined surface could be isolated. Within the extracted segment, a projection method was used to define the material orientation. Therefore, we investigated multiple printing angles, namely 0°, 30°, 45°, 60°, and 90°, conducted numerical simulations for each configuration, and subsequently compared the results with those obtained using the classical laminate theory (CLT). As illustrated in Figure 7d, the numerical results from the projection method almost perfectly coincided with the CLT results for all the tested angles. This agreement demonstrates that the projection method can be employed to assign shell element orientations that accurately reflect the actual fiber layout, thus yielding simulation outputs that closely match the theoretical calculations.

In conclusion, the numerical simulations and theoretical verifications conducted on this simplified model conclusively demonstrated the feasibility and accuracy of the projection method while providing valuable insights for its application to large-scale complex 3D-printed structures. This approach not only enables a precise definition of the material anisotropy within 3D-printed components but also provides critical technical support for accurate prediction of their mechanical performance.

## 4. Application to Mock-Up Ship

In this study, large-scale additive manufacturing (3D printing) technology was employed to comprehensively digitally design and produce a ship hull model measuring approximately 20 m in length. A vessel comprises several principal structural components, including the hull, deck, midsection, bottom, and aft sections.

**Figure 8:**
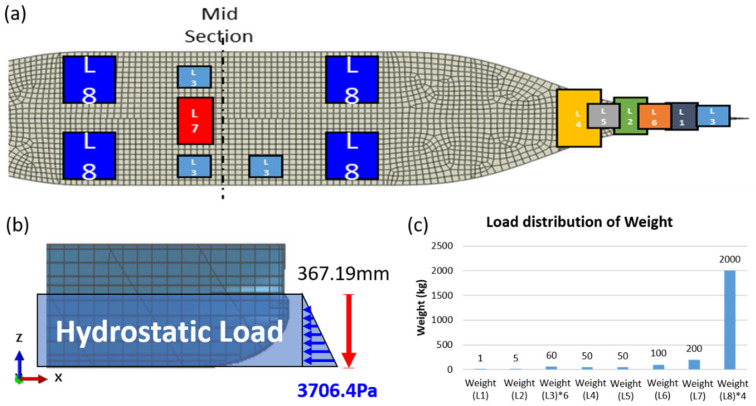
Load distribution of the Ship Model: (**a**) Load application zones illustrated on the hull structure; (**b**) Hydrostatic pressure applied to the side surface; (**c**) Weight values of individual concentrated loads distributed along the deck.

Before the manufacturing process, a numerical simulation was conducted on the ship structures using the finite-element software Abaqus. In the finite element simulation of the 3D-printed ship structure, appropriate boundary and loading conditions were defined to replicate a realistic service environment. The load distribution applied is illustrated in Figure 8. Concentrated and distributed weights were assigned to different regions of the ship model (L1–L8) to reflect the actual mass distribution observed during the experimental setup. Additionally, hydrostatic pressure was simulated as a vertical load of 3706.4 Pa at a depth of 367.19 mm to mimic the buoyancy during floating. This configuration replicates the combined effects of gravity, ballasts, and hydrostatic support under real conditions. The left end of the structure was constrained in the vertical (UY) and out-of-plane (UZ) directions (UY = UZ = 0), while the right end was fully fixed, restricting the displacements in all three directions (UX = UY = UZ = 0). The applied loads consisted of gravity (9.86 m s^−2^ acting downward), a maximum hydrostatic pressure of 0.037 MPa applied to the bottom surface, and eight concentrated loads distributed along the deck to simulate the operational conditions. Material properties were assigned based on the experimentally characterized mechanical behavior of the printed short-fiber-reinforced thermoplastic composites, as summarized in Table 1, to accurately capture their anisotropic mechanical behavior. Additionally, to correctly represent the fiber orientation within the simulation, a projection method was employed to map the local fiber directions based on the actual printing paths.

**Figure 9:**
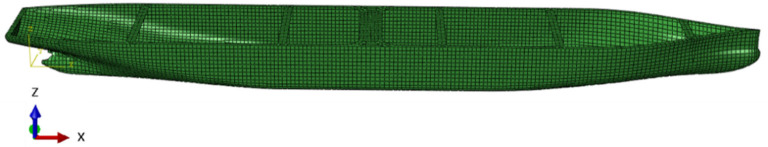
Finite element mesh of the 3D-printed ship structure. The simulation was performed using the ABAQUS software (Dassault Systèmes, Providence, RI, USA).

**Figure 10:**
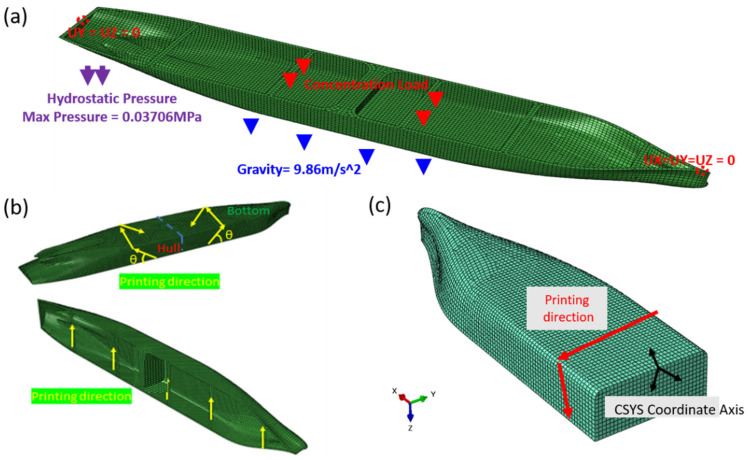
Overview of boundary conditions, printing strategy, and coordinate system setup: (**a**) boundary conditions, including gravity, hydrostatic pressure, and point loads; (**b**) direction of actual printing; and (**c**) method of setting material orientation. The simulation was performed using the ABAQUS software (Dassault Systèmes, Providence, RI, USA).

First, we compared the simulation results using isotropic and anisotropic material properties to evaluate how different simulation approaches affect the structural response. As shown in Figure 9, the entire hull structure was meshed with 10,044 shell elements. Among them, 9989 were 4-node linear quadrilateral elements (S4R), and 146 were 3-node linear triangular elements (S3R). This mesh configuration provided a good balance between capturing the geometric features and maintaining computational efficiency. To accurately represent the anisotropic material behavior resulting from the printing process, a local coordinate system (CSYS) was defined for each surface region of the model. The actual printing direction was used to determine the material orientation of each element by projecting it onto the inclined surface. These directions were implemented in Abaqus using element-wise orientation assignments. A visual summary of the boundary conditions, printing strategy, and coordinate system alignment is shown in Figure 10.

**Figure 11:**
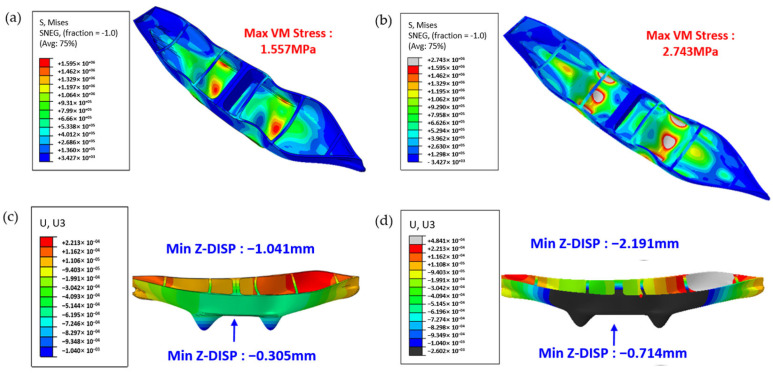
Finite element results comparing isotropic and anisotropic modeling approaches for the full-scale 3D-printed ship structure: (**a**) Isotropic simulation: von Mises stress distribution under the specified loading conditions, with maximum stress of 1.557 MPa; (**b**) Anisotropic simulation using projection-based fiber orientations, showing increased maximum stress of 2.743 MPa; (**c**) Isotropic model: predicted mid-span vertical displacement (Z direction) of −0.305 mm at the midpoint and −1.041 mm overall; (**d**) Anisotropic model: predicted displacement values increase to −0.714 mm (midpoint) and −2.191 mm overall, more closely matching experimental observations. The simulation was performed using the ABAQUS software (Dassault Systèmes, Providence, RI, USA).

As illustrated in Figure 11, simplifying the material as isotropic yields a maximum von Mises stress of only 1.56 MPa, whereas adopting an anisotropic model increases this value to 2.46 MPa. In terms of the Z-axis displacement, the isotropic model exhibits a minimum displacement of approximately −1.04 mm and a midpoint displacement of approximately −0.305 mm, whereas the anisotropic model yields a minimum displacement of −2.19 mm and a mid-point displacement of −0.714 mm. These findings underscore that neglecting the intrinsic anisotropy of composite materials can result in a significant underestimation of key mechanical indicators, particularly normal stresses and interlayer deformations. Only by thoroughly accounting for interlayer bonding, fiber orientation, and other anisotropic factors at the simulation stage can the mechanical behavior of a structure under real operating conditions be accurately captured, thereby providing a reliable foundation for subsequent ship structure design and safety assessments. This further indicates the considerable potential of the projection method for practical engineering simulation applications.

**Figure 12:**
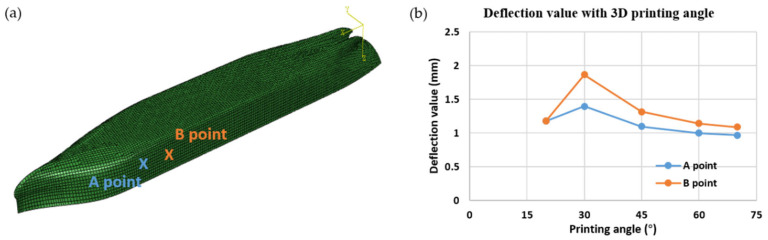
Variation in the deflection with the printing direction: (**a**) Isotropic simulation results-Von Max Stress; (**b**) Summary of experimental and predicted values. The simulation was performed using the ABAQUS software (Dassault Systèmes, Providence, RI, USA).

In the numerical analyses designed to examine the influence of printing orientation on hull deflection, we considered angles of 0°, 20°, 30°, 40°, 50°, 60°, and 70°. As shown in Figure 12, the results reveal a marked variation in the deflection values at points A and B in response to changes in the printing angle, indicating that the material deposition direction and interlayer bonding characteristics substantially affect the hull stiffness and overall stability. In the experimental phase of this study, a large-scale 3D-printed ship fabricated with a 45° printing angle will be floated in a water tank and subjected to a prescribed internal ballast load; the resulting deflections at monitoring points A and B will be recorded and rigorously benchmarked against the corresponding finite-element predictions.

**Figure 13:**
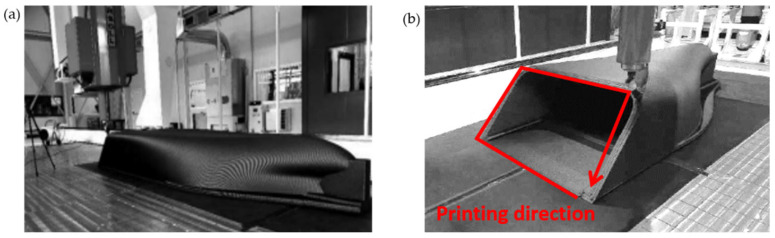
Ship CAD drawing and photo of the 3D printing process: (**a**) Printing process of 20-m-long ship model; (**b**) Printing direction of the 3D-printed ship.

In this study, a ship fabricated at a 45° printing angle was selected as the subject of investigation. This angle was chosen as a representative case that introduces a balanced combination of tensile and shear stress components in the printed structure. In composite material testing, a 45° fiber orientation is widely used to activate off-axis deformation modes and evaluate anisotropic behavior, particularly shear-dominated responses. Therefore, a 45° angle provides a mechanically meaningful scenario for validating the proposed method under nontrivial stress conditions. In addition, the 45° inclination allows continuous material deposition without requiring support structures and is compatible with the robotic printing configuration. While other angles, such as 30° or 60°, are also feasible, the 45° case offers a good compromise between mechanical sensitivity and manufacturing practicality, making it a suitable choice for validation. As shown in Figure 13, the ship was fabricated using deposition-based additive manufacturing, printing it upside down on a 45° inclined surface. During the manufacturing process, a high-capacity extruder capable of depositing material at approximately 150 lb h^−1^ was utilized, coupled with a nozzle diameter of 10 mm; each layer featured a width of approximately 18.8 mm and a thickness of 3 mm, with a printing orientation set at 45°. Upon completion of the printing phase, subsequent post-processing steps, such as surface polishing, coating, and targeted reinforcement, were performed to ensure overall structural integrity and enhanced mechanical properties. Compared with conventional shipbuilding techniques, this large-scale additive manufacturing approach reduces not only the production time but also the reliance on mold fabrication and labor-intensive processes, thereby laying a solid foundation for fabricating larger and more complex marine structures.

## 5. Deflection Measurement and Comparison

**Figure 14:**
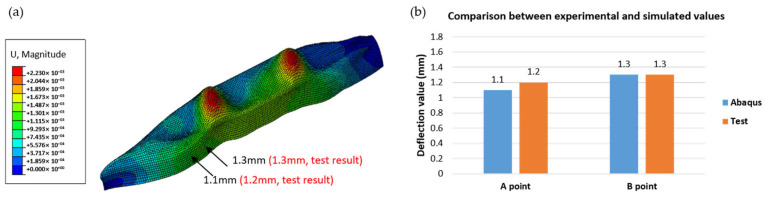
Comparison between experimental and simulated deflection results for 3D-printed ship structure. (**a**) Finite element prediction of vertical displacement (U3) under applied loading, with measurement points A and B annotated for deflection values. (**b**) Bar chart summarizing the measured and simulated mid-span deflections at points A and B. The simulation was performed using ABAQUS software (Dassault Systèmes, Providence, RI, USA).

After the 3D-printed ship was fabricated, experimental tests were conducted, and the results were compared with the simulation. The tests were performed in a laboratory environment at an ambient room temperature (approximately 22 °C). The printed ship was placed in a water tank and allowed to float freely to simulate hydrostatic support. No rigid constraints were imposed on the hull to minimize the artificial stiffness effects. Internal pressure was not applied; instead, deformation was induced by external gravity and distributed loads applied vertically at specific locations, as shown in Figure 8. These loading conditions were designed to replicate the real-world loading scenarios of a floating vessel underweight and with ballast. According to the simulation conditions, the ship was placed on water and subjected to a load. As illustrated in Figure 12a, two representative measurement points (A and B) were selected on the outer surface of the hull to monitor deformation during the loading process. Since the actual 3D- printing procedure was carried out at a 45°angle, we could compare the numerical findings with the experimental measurements under these conditions. As shown in Figure 14, a comparison of the deflection values (e.g., at points A and B) indicates that the discrepancy between the simulation and experimental data at 45° remains within 5%. The experimental deflection values were obtained from multiple repeated measurements at each point using a high-precision laser displacement sensor. The variation among the repeated readings remained within ±0.1 mm, lending confidence to the reported agreement between the simulation and experimental results. This consistency demonstrates that the proposed numerical model and analytical approach effectively capture real-world load and deformation behaviors. Consequently, we infer that as long as the material modeling assumptions and simulation procedures remain consistent, a credible prediction of the mechanical performance can also be achieved for other angles or further process refinements.

## 6. Conclusions

In this study, we developed a projection-based methodology for accurately analyzing large polymer composite structures fabricated using FDM-based 3D printing. By defining local fiber orientations based on actual printing paths, the method enables the precise modeling of anisotropic mechanical behavior in finite element simulations. The projection framework was validated through theoretical derivation and experimental comparison using a large-scale 3D-printed mockup, and excellent agreement was observed between the predicted and measured deformations. The direction-dependent mechanical properties, including tensile and shear data, were characterized and integrated into the simulation. The results demonstrate the effectiveness and practical applicability of the proposed method in improving the simulation accuracy of anisotropic, additively manufactured composite components.

Although this approach shows good predictive capability, it currently assumes simplified printing paths on segmented surfaces with smoothly varying geometries. Highly irregular structures or components with significant inter-layer property gradients may challenge the stability and accuracy of the projection algorithm. In addition, the material behavior is modeled as transversely isotropic; extending the method to orthotropic or multi-phase systems will require further development.

Future studies will focus on expanding the methodology to more complex geometries and multiaxial printing paths. Further investigation into the effects of different printing angles and fiber orientations may provide deeper insights into the process–structure–property relationships. The framework can also be adapted to multi-material or hybrid composites, where variations in stiffness across layers and interfaces introduce new modeling challenges. These extensions are expected to enhance the generality and scalability of the proposed approach for large-scale additive manufacturing applications.

## Figures and Tables

**Table 1:** Summary of the experimental results for ABS/CF20.

	Stiffness				Strength		
Direction	0°	90°	-	10°	0°	90°	10°
Symbol	$E_{x}$	$E_{y}$	$\nu_{xy}$	$G_{y}$	X	Y	S
Properties	9.80	3.23	0.320	1.17	81.1	20.2	10.9
Standard Deviation	0.320	0.117	0.006	0.061	3.28	1.88	0.712

## Data Availability

The original contributions presented in this study are included in the article. Further inquiries can be directed to the corresponding author.
